# Evidence of *Helicobacter pylori* heterogeneity in human stomachs by susceptibility testing and characterization of mutations in drug-resistant isolates

**DOI:** 10.1038/s41598-024-62200-1

**Published:** 2024-05-27

**Authors:** Jahirul Md Islam, Yukari Yano, Aoi Okamoto, Reimi Matsuda, Masaya Shiraishi, Yusuke Hashimoto, Nanaka Morita, Hironobu Takeuchi, Narufumi Suganuma, Hiroaki Takeuchi

**Affiliations:** 1 Department of Medical Laboratory Sciences, Health, and Sciences, International University of Health and Welfare Graduate School, Chiba, Japan; 2 Kochi Medical School, Kochi Community Medical Support Center, Kochi, Japan; 3 Gifu Prefectural General Medical Center, Gifu, Japan; 4 Department of Occupational and Environmental Medicine, Kochi Medical School, Kochi, Japan

**Keywords:** Genetics, Molecular biology, Gastroenterology, Health care, Medical research

## Abstract

Heterogeneity of *Helicobacter pylori* communities contributes to its pathogenicity and diverse clinical outcomes. We conducted drug-susceptibility tests using four antibiotics, clarithromycin (CLR), amoxicillin (AMX), metronidazole and sitafloxacin, to examine *H. pylori* population diversity. We also analyzed genes associated with resistance to CLR and AMX. We examined multiple isolates from 42 Japanese patients, including 28 patients in whom primary eradication with CLR and AMX had failed, and 14 treatment-naïve patients. We identified some patients with coexistence of drug resistant- and sensitive-isolates (drug-heteroR/S-patients). More than 60% of patients were drug-heteroR/S to all four drugs, indicating extensive heterogeneity. For the four drugs except AMX, the rates of drug-heteroR/S-patients were higher in treatment-naïve patients than in primary eradication-failure patients. In primary eradication-failure patients, isolates multi-resistant to all four drugs existed among other isolates. In primary eradication-failure drug-heteroR/S-patients, CLR- and AMX-resistant isolates were preferentially distributed to the corpus and antrum with different minimum inhibitory concentrations, respectively. We found two mutations in PBP1A, G591K and A480V, and analyzed these in recombinants to directly demonstrate their association with AMX resistance. Assessment of multiple isolates from different stomach regions will improve accurate assessment of *H. pylori* colonization status in the stomach.

## Introduction

*Helicobacter pylori* is a highly genetically diverse, gram-negative, microaerophilic pathogenic bacterium that colonizes the stomach^1,2^. *H. pylori* is thought to cause gastritis, peptic ulcer disease, gastric adenocarcinoma, and gastric lymphoma^3–5^. *H. pylori* is also involved in the development of extra-gastrointestinal diseases, including iron deficiency anemia, immune thrombocytopenia, and acute coronary syndromes^6^. *H. pylori* is a group I carcinogen that is responsible for more than 80% of gastric cancers and is the fourth leading cause of cancer-related deaths worldwide^7,8^. The prevalence of *H. pylori* infection is 40–75% in the global population; approximately 70% in developing countries and 25–50% in developed countries^9^. In 2017, *H. pylori* was included in the World Health Organization’s “priority list of antibiotic-resistant bacteria” and was ranked top among the most common community-acquired infections^10^.

The efficacy of *H. pylori* eradication therapy is diminishing because of the rise of antimicrobial resistance. Clarithromycin (CLR), commonly used in triple therapy regimens, is an effective antibiotic for the treatment of *H. pylori* infection^11^; however, eradication failure has increased with the emergence of CLR-resistant strains. The first reported eradication rate for primary standard triple therapy in Japan was just over 85%. This then declined because of the spread of CLR-resistant *H. pylori*^12^. A recent meta-analysis of data from 54 countries showed a CLR-resistant strain prevalence of 27.5%. This resistance rate increased from 24.3% in 2010–2017 to 32.1% in 2018–2021^13^. The differences in reported rates of CLR resistance result from differences in antibiotic use, patient compliance, access to healthcare, bacterial evolution and mutation, and other factors. Point mutations in *23S rRNA*, specifically A2142G and A2143G in domain V, are associated with CLR resistance of *H. pylori*^14–16^. These mutations induce structural changes in the ribosomal binding site and reduce the affinity of CLR to *23S rRNA* in the *50S rRNA* subunit. Other rarely reported point mutations in *23S rRNA* include; A2115G, T2117C, G2141A, T2182C, G2224A, C2245T, T2289C, C2611A, and T2717C^17–19^. Of these, A2115G, T2117C, G2141A, G2224A, C2245T, and T2289C mutations have not been confirmed. However, T2182C, C2611A, and T2717C are associated with low levels of CLR resistance^20^.

Amoxicillin (AMX), a β-lactam antibiotic, is widely used to treat *H. pylori* infection. Mutations in the genes encoding penicillin-binding proteins can reduce affinity for AMX, resulting in AMX resistance^21,22^, although the prevalence of AMX-resistant strains is comparatively low^23^. A meta-analysis demonstrated that the primary resistance rate of AMX was approximately 3% in the Asia–Pacific region^24^. However, the prevalence of AMX-resistant strains is gradually increasing because of mutations in PBP1A^25–27^. Primary AMX resistance rates have recently reached 13% in Japan^28^. The resistance rates vary by region, but there is a worrying global trend of increasing drug resistance^23^. A minimum inhibitory concentration (MIC) of > 0.125 μg/mL defines AMX resistance (European Committee on Antimicrobial Susceptibility Testing)^29^, and *H. pylori* with a MIC of ≥ 0.5 μg/mL is considered AMX-high resistance (AMX^HR^)^30^. Eradication failure rates were significantly higher in cases of AMX^HR^ compared with cases with lower AMX resistance^30^. The use of MIC ≥ 0.5 μg/mL has been recommended as an index for judging therapeutic efficacy.

The treatment of *H. pylori* infection using CLR and AMX as primary eradication therapy has been widely employed. The success rate of primary eradication therapy that includes vonoprazan (a potassium-competitive acid blocker) instead of a proton pump inhibitor is currently acceptable, but the eradication failure rate is increasing because of the emergence of CLR-resistant and AMX-resistant *H. pylori* strains. In Japan, patients can receive insurance-covered primary eradication therapy without susceptibility testing. Susceptibility tests usually involve the laboratory examination of single isolate from patients.

*H. pylori* exhibits high genetic diversity, with DNA sequence polymorphism ranging from 2.7 to 8.0%^31–33^. Intraspecific genetic diversity produces diverse phylogenetic populations. This genetic diversity is generated through multiple mechanisms, including spontaneous point mutations, recombination with other bacteria, and intragenomic rearrangements involving mobile genetic elements or repetitive DNA sequences. These characteristics of *H. pylori* affect the heterogeneity of the community in the stomach. However, previous reports have commonly focused on findings derived from a single isolate obtained from a patient. The adaptability and flexibility of *H. pylori* allows the community to continuously adapt to changes in the gastric environment, leading to persistent infection and the emergence of considerable heterogeneity^2^. *H. pylori* diversity is an important phenomenon to consider when studying the pathogenesis of this bacterium and developing new treatments for *H. pylori*-associated diseases^34^.

The prevalence of heteroresistance to various antibiotics has been reviewed as impacting the reduced efficacy of eradication therapy with silent resistant isolates that were not discovered in clinical laboratory due to the low number of isolates tested^35,36^. A systematic review coupled with meta-analysis showed that the heteroresistance rate was 6.8% for CLR and 13.8% for metronidazole (MNZ)^37^. There is little literature on heteroresistance to AMX and sitafloxacin (STFX), and the rates of 3–19.6% for AMX and 0–18.2% for STFX are reported in unpublished data. However, these studies consider the heterogeneity of *H. pylori* in the stomach, usually being limited to the analysis of two or three isolates per patient, which is still controversial. The *H. pylori* community consisting of isolates with different properties adapts to the gastric environment as persistent infection. The heterogeneity of *H. pylori* in the stomach, particularly in primary eradication-failed patients, is uncertain. We therefore identified the heterogeneity of *H. pylori* in the stomach by susceptibility testing using multiple isolates from patients to characterize drug-resistant isolates in the *H. pylori* community. Furthermore, we identified mutations associated with CLR and AMX antibiotic resistance.

## Materials and methods

### Patients and samples

This study was approved by the Ethics Committee of Kochi University (approval number 27–35) and human samples were used in accordance with the guidelines of Kochi Medical University Hospital and the Declaration of Helsinki. Written informed consent was obtained from all enrolled patients. Multiple isolates were collected from 42 Japanese patients who underwent upper gastrointestinal endoscopy at Kochi Medical University Hospital. The 42 patients consisted of 17 males and 25 females aged 35–89 years (Table 1). Twenty-eight patients in whom primary eradication therapy with CLR and AMX failed were assigned to an eradication-failure group and 14 patients who had never received antibiotic eradication therapy were assigned to a treatment-naïve group. To assess the heterogeneity of *H. pylori* community within the stomach, 2–3 biopsy specimens were endoscopically collected from distinct regions (antrum, corpus, and the area close to a tumor region (tumor)). The specimens were subjected to histopathological analysis and to homogenization for bacterial culture. The homogenized biopsies were spread on selective plates for *H. pylori* (Vi HELICO AGAR, Eiken Chemical Co. Ltd, Japan) as primary selection step. We carefully obtained multiple colonies by detailed observations of individual properties such as growth speed, colony size and color, etc. in the primary selection step. The isolates were identified by gram-staining and PCR as previously described^38^ (Supplementary Table S1). At least five isolates were collected from each biopsy specimen of the stomach (antrum, corpus, and tumor). The multiple isolates used in this study per patient (Table 1). A total of 512 isolates (231, 241, and 40 isolates from antrum, corpus, and tumor, respectively) were isolated, consisting of 319 and 193 isolates from primary eradication-failed and treatment-naïve patients, respectively.

**Table 1:** List of patients’ information.

Patients	Isolates	Primary eradication treatment	Age	Sex	Heterogeneity				Multi-resistance^a^
					CLR	AMX	MNZ	STFX	
E24	12	Failure	57	M	■	○	■	■	4
E7	12	Failure	61	M	■	■	○	○	4
E14	12	Failure	45	M	■	○	■	○	4
E50	12	Failure	68	F	■	○	■	○	4
E39	10	Failure	56	M	■	□	■	■	3
E22	12	Failure	55	F	■	□	■	■	3
E46	12	Failure	69	F	■	□	■	■	3
E10	12	Failure	56	F	■	□	■	○	3
E45	12	Failure	54	F	■	□	■	○	3
E21	12	Failure	59	F	■	□	○	■	3
E33	11	Naïve	38	F	■	□	○	○	3
H14	18	Naïve	73	F	■	□	○	○	3
H11	18	Naïve	87	F	■	□	○	○	3
E1	12	Failure	35	F	■	□	■	□	2
E2	12	Failure	43	F	■	□	■	□	2
E12	12	Failure	48	F	■	□	■	□	2
E19	12	Failure	48	F	■	□	■	□	2
E32	12	Failure	42	F	■	□	■	□	2
E34	12	Failure	56	F	■	□	■	□	2
E36	06	Failure	56	F	■	□	■	□	2
H20	12	Failure	64	F	■	□	□	■	2
E23	12	Failure	67	M	■	□	□	■	2
E13	12	Failure	76	M	■	□	○	□	2
E20	06	Failure	43	M	■	□	○	□	2
E18	12	Naïve	42	F	■	□	○	□	2
E44	12	Naïve	58	F	■	□	□	○	2
H18	12	Failure	53	M	■	□	□	○	2
H24	18	Failure	80	M	■	□	□	○	2
H19	12	Failure	54	M	■	○	□	□	2
E28	12	Failure	61	F	○	□	■	□	2
E8	11	Naïve	63	M	□	□	○	○	2
E30	12	Failure	60	F	○	□	○	□	2
E41	12	Naïve	54	F	■	□	□	□	1
E15	11	Naïve	52	F	○	□	□	□	1
E49	12	Failure	60	F	○	□	□	□	1
H2	15	Naïve	69	M	□	□	○	□	1
H15	06	Naïve	87	M	□	□	○	□	1
H16	12	Naïve	54	M	□	□	○	□	1
H23	18	Naïve	58	M	□	□	○	□	1
H22	24	Naïve	74	F	□	□	□	○	1
E9	03	Failure	42	M	□	□	□	□	0
H9	13	Naïve	68	M	□	□	□	□	0

*CLR* clarithromycin, *AMX* amoxicillin, *MNZ* metronidazole, *STFX* sitafloxacin, *Failure* primary eradication-failure, *Naïve* treatment-naïve.

■, Drug-homoR-patients; ○, Drug-heteroR/S-patients; □, Drug-homoS-patients.

^a^Number of antibiotics for multi-resistant isolates.

### Bacterial strains and culture conditions

#### Helicobacter pylori

*H. pylori* strains, J99, 26695 and HPK5, clinical isolates and engineered recombinants, were used in this study (Table 2). *H. pylori* was grown on Brucella broth (Becton, Dickinson and Company, USA) agar plates (BB-plates) supplemented with 10% horse serum, vancomycin (10 μg/mL), and 1.4% agar (Wako Pure Chemical, Japan), at 37 °C under 10% CO_2_ (Sanyo CO_2_ incubator, Japan). Brucella broth supplemented with 10% horse serum (BB-liquid) was used when appropriate. Isolates were stored at − 80 °C until use.

**Table 2:** Bacterial strains, plasmids, and MIC values (µg/mL).

Strain or plasmid	Mutation/description		MIC of AMX/CLR		Source
Donor DNA					
AMX					
E7AG3	N504D, I515M, N562Y, S589G, G591K^a^, T593G, G595S	*pbp1A*	0.75	AMX	This study
E50AG3	S414R, A480V^a^, N504D, T556S, S589G, 595_596 Insertion G		0.5		This study
E7BG5	N504D, I515M, N562Y, S589G, T593G, G595S		0.5		This study
E50BG4	S414R, N504D, T556S, S589G, 595_596 Insertion G		0.38		This study
CLR					
E23AG1	T1644C, A1738G, A2121G, G2126A, T2130C, A2143G, T2182C, T2244C	*23S rRNA*	48	CLR	This study
H11BG3	A1738G, A2121G, G2126A, T2130C, A2143G, T2182C, T2244C		6		This study
Recipients					
J99	Drug sensitive strains in 4 antibiotics (CLR, AMX, MNZ, STFX) No A1738G mutation in 23S rRNA No A480V and G591K mutations in PBP1A		< 0.016	AMX/CLR	United States
26695			< 0.016		United Kingdom
HPK5			< 0.016		Japan
*E. coli*					
DH5α	Used in transformation				Nippon gene
Plasmids					
pE7/TOPO2.1	TOPO2.1 vector harboring 905 bp fragment with G591K mutation				This study
pE50/TOPO2.1	TOPO2.1 vector harboring 905 bp fragment with A480V mutation				This study
pE7BG5/TOPO2.1	TOPO2.1 vector harboring 905 bp fragment without G591K mutation				This study
pE50BG4/TOPO2.1	TOPO2.1 vector harboring 905 bp fragment without A480V mutation				This study
Recombinants					
AMX					
E7. J99	S414R, N504D, I515M, N562Y, S589G, G591K^a^, T593G, G595S	*pbp1A*	1.5	AMX	This study
E7. 26695	S414R, D479E, N504D, I515M, N562Y, S589G, G591K^a^, T593G, G595S		0.25		This study
E7. HPK5	S414R, S558T, N562Y, K590Q, G591K^a^, T593G, G595S		0.25		This study
E50. J99	S414R, A480V^a^, N504D, M515I, T556S, S589G, 595_596 Insertion G		0.19		This study
E50. 26695	S414R, A480V^a^, N504D, M515I, T556S, S589G, 595_596 Insertion G		0.19		This study
E50. HPK5	S414R, A480V^a^, M515I, T556S, S558T, 595_596 Insertion G		0.094		This study
E7BG5. J99	N504D, I515M, N562Y, S589G, T593G, G595S		0.023		This study
E7BG5. 26695	E448G, F473S, N504D, I515M, N562Y, S589G, T593G, G595S		0.047		This study
E7BG5. HPK5	N504D, I515M, N562Y, S589G, T593G, G595S		0.032		This study
E50BG4. J99	K315E, D336E, S414R, N504D, T556S, S589G, 595_596 Insertion G		0.032		This study
E50BG4. 26695	S414R, N504D, T556S, S589G, 595_596 Insertion G		0.023		This study
E50BG4. HPK5	S414R, N504D, T556S, S589G, 595_596 Insertion G		0.023		This study
CLR					
E23. J99	T1644C, A1738G	*23S rRNA*	< 0.016	CLR	This study
H11. J99	A1738G		< 0.016		This study

*CLR* clarithromycin, *AMX* amoxicillin, *MNZ* metronidazole, *STFX* sitafloxacin.

^a^Mutations (A480V and G591K) found are unique to only amoxicillin high-resistant isolates.

#### Escherichia coli

*E. coli* DH5α (Nippon gene, Japan) was used to construct plasmids. Bacteria were cultured on Luria–Bertani broth (Nacalai Tesque, Japan) agar plates (LB-plates) supplemented with ampicillin (Amp) (50 μg/mL) and 1.4% agar at 37 °C under aerobic conditions.

### Drug-susceptibility tests

The 512 isolates from the 42 patients were subjected to drug-susceptibility tests with four antibiotics, CLR, AMX, metronidazole (MNZ) and sitafloxacin (STFX), to obtain MICs. The E-test was used for CLR, AMX and MNZ, and the agar dilution method was used for STFX. Briefly, well-grown isolates were suspended in 1 mL of BB-liquid (to absorbance > 1.0 at OD_600_) and inoculated onto BB-plates with sterile cotton swabs. E-test strips (Biomerieux, Japan) were then placed on the plates according to manufacturer’s instructions. These plates were incubated at 37 °C for 3–5 days. MIC values were determined, and antibiotic resistance was defined as; MIC ≥ 1 μg/mL for CLR, > 0.125 μg/mL for AMX, ≥ 16 μg/mL for MNZ and ≥ 0.5 μg/mL for STFX. Based on the drug-susceptibility tests, the 42 patients were classified into three categories: drug-heteroResistant/Sensitive (heteroR/S)-patients (in whom resistant and sensitive isolates coexist in the stomach), drug-homoresistant (homoR)-patients (in whom only resistant isolates are present in the stomach), and drug-homosensitive (homoS)-patients (in whom only sensitive isolates are present in the stomach).

### PCR and cycle sequencing

Bacterial genomic DNA was extracted using a QIAprep Spin Miniprep Kit (QIAGEN, Germany) and used to PCR amplify *23S rRNA*^39^ and *pbp1A*^28^. The PCR conditions and primers used are listed in Supplementary Table S1. The PCR amplicons were then purified using a Fast Gene Extraction Kit (Nippon Genetics Co. Ltd., Japan) and the expected product sizes of 1093 bp for *23S rRNA* and 905 bp for *pbp1A* were confirmed. Amplicons were cycle sequenced using Big Dye Terminator (Applied Biosystems, USA) and a specific primer in 20 μL reaction mixtures. Ten μL of Xterminator solution and 45 μL of SAM solution (Applied Biosystems, USA) were added to the mixture, vigorously mixed (vortexed for 15 min), and then the mixture was applied to a DNA sequencer (3500xL Genetic Analyzer, 24chRUO, HITACHI, Japan) according to manufacturer’s instructions.

### Mutation analysis

We performed mutation analysis of *23S rRNA* in 116 randomly selected isolates from 10 patients, including 65 isolates from all 5 CLR-heteroR/S-patients, 24 isolates from 2 CLR-homoR-patients, and 27 isolates from 3 CLR-homoS-patients. For amino acid analysis of PBP1A, 87 isolates were analyzed, including 48 isolates from all 4 AMX-heteroR/S-patients, 12 isolates from 1 AMX-homoR-patient and 27 isolates from 3 AMX-homoS-patients. The sequence of double strand was performed more than two times for each target region and the alignment was analyzed to confirm the homology by the Basic Local Alignment Search Tool (BLAST). BLASTn and BLASTp were used for nucleotide and amino acid sequences analyses based on Swiss-Prot database, respectively. Four (HPU27270.1, J99, 26695, and HPK5) and five (1061, 69A, J99, 26695, and HPK5) strains were used as reference strains for CLR and AMX, respectively.

### Construction of plasmids

The bacterial strains and plasmids used are listed in Table 2. To identify mutations associated with AMX resistance, we constructed two plasmids (pE7/TOPO2.1 and pE50/TOPO2.1) for use in homologous recombination to generate six recombinants based on three *H. pylori* strains (J99, 26695 and HPK5). We used genomic DNA of two AMX-resistant isolates (E7AG3 and E50AG3) that possess G591K and A480V mutations in PBP1A, respectively, which are thought to be associated with AMX resistance. Briefly, PCR was performed using genomic DNA of the two isolates. Each amplicon was ligated into pCR^TM^2.1-TOPO® using a TOPO Cloning kit (Invitrogen, USA) to construct pE7/TOPO2.1 and pE50/TOPO2.1. Aliquots of the ligation mixture were used to transform *E. coli* DH5α, which were then spread on pre-warmed LB-plates containing 50 µg/mL Amp, X-Gal, and IPTG, and incubated overnight at 37 °C. We selected several white colonies and performed PCR to confirm the plasmids harboring the PCR fragments. Finally, PCR fragments amplified from pE7/TOPO2.1 and pE50/TOPO2.1 were confirmed by sequencing. We also constructed two plasmids to confirm involvement of the mutation associated with AMX resistance. We used genomic DNA of two further AMX-resistant isolates (E7BG5 and E50BG4), which possess the same amino acid sequences as E7AG3 and E50AG3 except they do not contain the G591K and A480V mutations. According to the method described above, two plasmids (pE7BG5/TOPO2.1 and pE50BG4/TOPO2.1) were constructed. These four plasmids were used for homologues recombination to generate 12 recombinants.

### Homologous recombination

We performed homologous recombination to produce recombinants possessing the PBP1A G591K or A480V mutations using AMX-sensitive strains without such mutations (J99, 26695 and HPK5), as described previously^40^. Briefly, well-grown strains on BB-plates were inoculated into BB-liquid media to prepare high-concentration bacterial suspensions. An aliquot of plasmid DNA was added to 50 μL of bacterial suspension and dropped onto a BB-plate and incubated at 37 °C for 4–5 h. The drop was then spread using a sterile cotton swab and cultured for a few days. The bacteria that grew on the BB-plate were harvested, spread onto selective BB-plates supplemented with various concentrations of Amp, and cultured at 37 °C until colonies emerged. A few colonies for each of the 12 recombinants were subjected to the AMX-susceptibility test to determine MIC values. The mutations in PBP1A were confirmed by sequencing (Table 2).

Instead of plasmids, purified PCR products from E23AG1 and H11BG3 isolates were used in homologues recombination with CLR-sensitive strain J99 to determine whether the A1738G mutation found contributes to CLR resistance. However, we did not obtain recombinants as there was no growth on CLR plates. Therefore, using several colonies grown on BB-plates without CLR, we performed PCR-based single nucleotide polymorphism (SNP) designed to select the recombinants that exchanged for A1738G. Finally, recombinants with a sequencing-confirmed A1738G mutation were subjected to the CLR-susceptibility test.

### Statistical analysis

Statistical analyses were conducted using SPSS statistical software package version 25.0 and *p* value ≤ 0.05 was considered statistically significance. The MIC values were validated by non-parametric Wilcoxon tests with medians and interquartile ranges.

## Results

### Drug-susceptibility testing of 512 isolates from 42 patients

**Figure 1:**
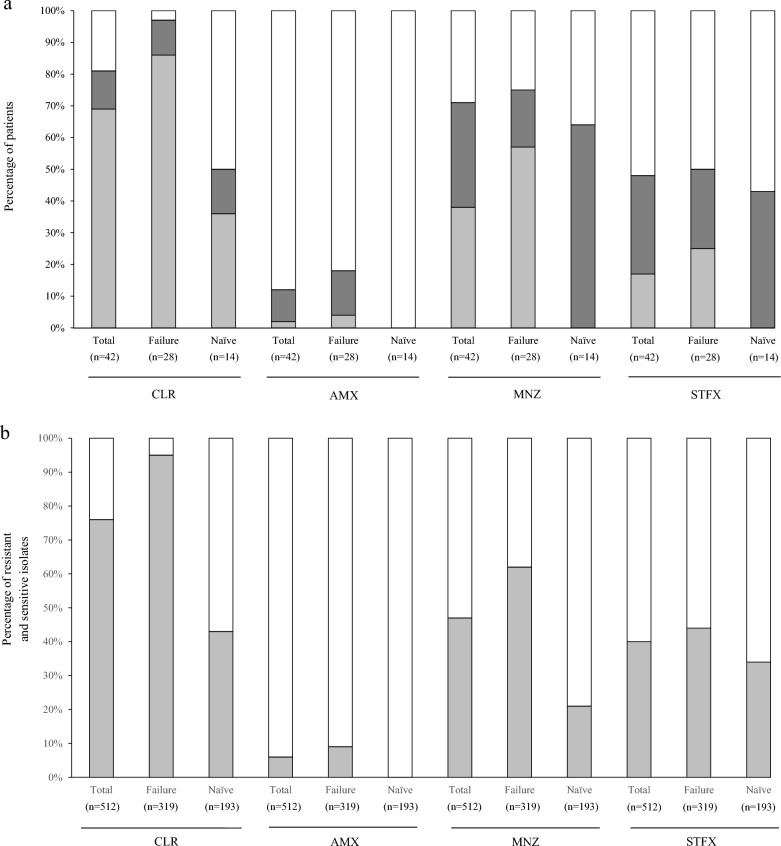
The proportions of patients and isolates based on drug-susceptibility test with four antibiotics. (**a**) The 42 patients are classified into three categories; drug-heteroR/S-patients, drug-homoR-patients and drug-homoS-patients. For CLR, the proportion of CLR-homoR-patients is considerable high in the primary eradication-failure group. CLR-homoS-patients dominate in the treatment-naïve group. CLR-heteroR/S-patients are slightly higher in treatment-naïve group than primary eradication-failure group. For AMX, AMX-homoR-patients and AMX-heteroR/S-patients are found in only primary eradication-failure group. For MNZ, MNZ-homoR-patients are found in only primary eradication-failure group and MNZ-heteroR/S-patients dominate in treatment-naïve group. For STFX, STFX-heteroR/S-patients are slightly higher in treatment-naïve group than primary eradication-failure group. STFX-homoR-patients are found in only primary eradication-failure group and STFX-homoS-patients dominate in two groups. light grey square, Drug-homoR-patients; grey square, Drug-heteroR/S-patients; white square, Drug-homoS-patients. (**b**) Total 512 isolates from 42 patients show the rates of resistant and sensitive isolates in 4 drugs. For CLR, the proportion of resistant isolates is high in primary eradication-failure group but nearly equal in treatment-naïve group. For AMX, resistant isolates are detected in only primary eradication-failure group, however, sensitive isolates dominate in the two groups. For MNZ, the proportion of sensitive isolates is small preponderance in primary eradication-failure group and sensitive isolates dominate in treatment-naïve group. For STFX, the proportion of sensitive isolates dominate in the two groups. light grey square, resistant isolates; white square, sensitive isolates. *Failure* primary eradication-failure, *Naïve* treatment-naïve, *CLR* clarithromycin, *AMX* amoxicillin, *MNZ* metronidazole, *STFX* sitafloxacin.

Multiple isolates from each stomach of 42 patients were evaluated by drug-susceptibility tests with four antibiotics to demonstrate community heterogeneity. The drug-susceptibility test clarified the coexistence of drug-resistant and drug-sensitive isolates in a stomach. For CLR, AMX, MNZ and STFX, 12%, 10%, 33% and 31%, respectively, of all 42 patients were drug-heteroR/S-patients. Sixty-four percent of the 42 patients were drug-heteroR/S for at least one of the four drugs. Drug-homoR-patients and drug-homoS-patients were observed and are summarized in Fig. 1a and Table 1. Of the 28 patients in whom primary eradication with CLR and AMX failed, 11%, 14%, 18% and 25% were drug-heteroR/S for CLR, AMX, MNZ and STFX, respectively. Of the 14 treatment-naïve patients, 14%, 0%, 64% and 43% were drug-heteroR/S for CLR, AMX, MNZ and STFX, respectively. Fifty-four percent and 86% of patients in the eradication-failure group and the treatment-naïve group, respectively, were drug-heteroR/S for at least one of the four drugs.

In the eradication-failure group 86% of patients were CLR-homoR. In the treatment-naïve group 36% were CLR-homoR-patients and 50% were CLR-homoS-patients. There was a similar prevalence of CLR-heteroR/S-patients in these two groups. AMX-homoR-patients and AMX-heteroR/S-patients were only observed in the eradication-failure group, indicating the consequence of primary eradication-failure. MNZ-homoR-patients were the most common patients in the eradication-failure group (at 57%) and MNZ-heteroR/S-patients were most common in the treatment-naïve group (at 64%). STFX-homoS-patients were the most common patients in the eradication-failure group (at 50%) and treatment-naïve group (at 57%). STFX-homoR-patients were only observed in the eradication-failure group. The susceptibility test using multiple isolates revealed that the infection rate of patients with drug-resistant isolates (drug-heteroR/S-patients and drug-homoR-patients) was higher than that for drug-homoR-patients, regardless of eradication treatment (Fig. 1a, Table 3). The evaluation of susceptibility test using a single isolate may not reflect the exact status of *H. pylori* infection in the human stomach. These findings emphasizes that susceptibility test using multiple isolates is importance and adequate method to evaluate the status of *H. pylori* infection in the stomach. Furthermore, 4, 9 and 19 patients were infected with isolates multi-resistant to 4, 3 and 2 drugs, respectively (Table 1). All four patients with isolates multi-resistant to all four drugs belonged to the eradication-failure group. The isolates multi-resistant to all four drugs coexisted with other isolates in the stomach.

**Table 3:** The infection rates of drug-homoR-patients and drug-heteroR/S-patients.

	Drug-homoR			Drug-heteroR/S			Drug-homoR and -heteroR/S		
	Total	Failure	Naïve	Total	Failure	Naïve	Total	Failure	Naïve
	(n = 42)	(n = 28)	(n = 14)	(n = 42)	(n = 28)	(n = 14)	(n = 42)	(n = 28)	(n = 14)
	%	%	%	%	%	%	%	%	%
CLR	69	86	36	12	11	14	81	97	50
AMX	2	4	0	10	14	0	12	18	0
MNZ	38	57	0	33	18	64	71	75	64
STFX	17	25	0	31	25	43	48	50	43
1/4 drugs^a^	71	89	36	64	54	86	95	96	93

*CLR* clarithromycin, *AMX* amoxicillin, *MNZ* metronidazole, *STFX* sitafloxacin, *Failure* primary eradication-failure, *Naïve* treatment-naïve.

^a^At least one of four drugs (CLR, AMX, MNZ, STFX).

CLR-resistant isolates accounted for 304 of the 319 isolates (95%) from eradication-failed patients and 83 of the 193 isolates (43%) from treatment-naïve patients (Fig. 1b). There were 29 (9%) AMX-resistant isolates in the eradication-failed patients and 0 (0%) in the treatment-naïve patients. For four drugs, AMX-sensitive isolates were most common among all 512 isolates, which is consistent with previous reports^28^. For MNZ, there were 198 (62%) and 41 (21%) resistant isolates in eradication-failed patients and treatment-naïve patients, respectively. Interestingly, MNZ-heteroR/S-patients were the most common patients (approximately 64%) among the treatment-naïve patients, but the prevalence of MNZ-resistant isolates was relatively low at 21%, indicating that MNZ-sensitive isolates dominated in MNZ-heteroR/S-patients. There were 139 (44%) and 66 (34%) STFX-resistant isolates in eradication-failed patients and treatment-naïve patients, respectively. STFX-sensitive isolates predominated in the two groups.

### Drug-heteroR/S-patients

**Figure 2:**
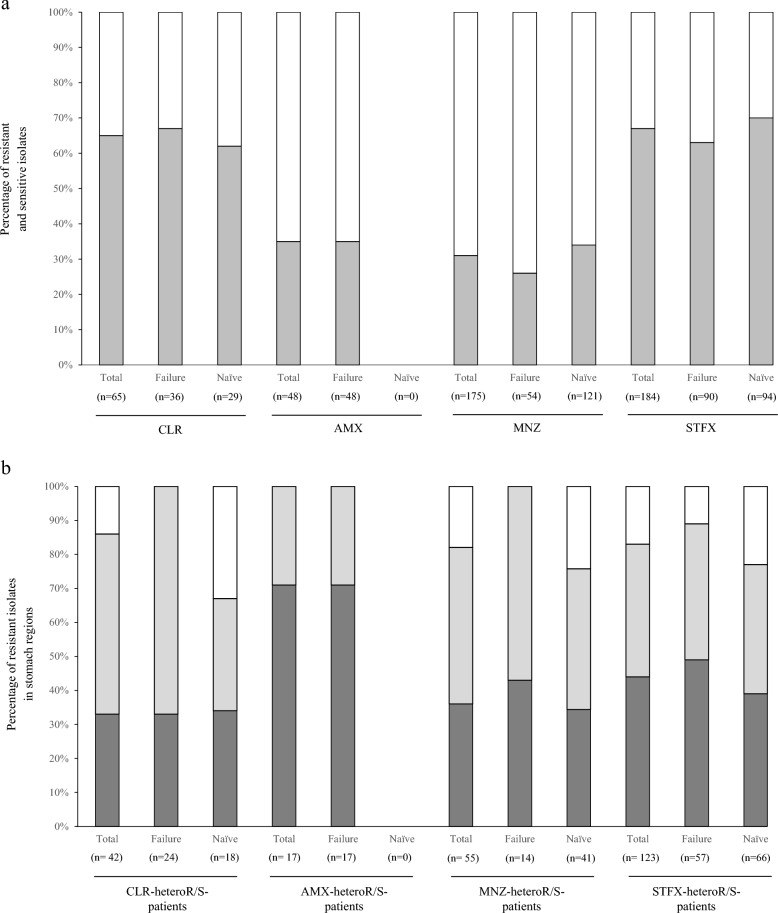
The characterization of isolates in the drug-heteroR/S-patients on four antibiotics. (**a**) The proportions of resistant and sensitive isolates in the drug-heteroR/S-patients. In CLR-heteroR/S-patients, resistant isolates dominate in the two groups. AMX-heteroR/S-patients are observed in only primary eradication-failure group, and resistant isolates are less than 40%. In MNZ-heteroR/S-patients, the proportions of sensitive isolates dominate in the two groups. Conversely, the proportions of resistant isolates dominate in the two groups of STFX-heteroR/S-patients. light grey square, resistant isolates; white square, sensitive isolates. (**b**) The distribution of resistant isolates in stomach regions of the drug-heteroR/S-patients. CLR-resistant isolates dominantly occupy in the corpus of the primary eradication-failed patients; however, the distribution is even across the stomach regions in treatment-naïve patients. Conversely, AMX-resistant isolates dominate in antrum of the primary eradication-failed patients. MNZ-resistant and STFX-resistant isolates are almost equivalent distribution across the different stomach regions, regardless of primary eradication treatment. grey square, antrum; light grey square, corpus; white square, tumor. *Failure* primary eradication-failure, *Naïve* treatment-naïve, *CLR* clarithromycin, *AMX* amoxicillin, *MNZ* metronidazole, *STFX* sitafloxacin.

We performed detailed analysis on drug-heteroR/S-patients in which both drug-resistant and drug-sensitive isolates coexist in the stomach and these results were summarized (Fig. 2a, Table 4). A total of 65 isolates were isolated from all 5 CLR-heteroR/S-patients, consisting of 3 eradication-failed patients and 2 treatment-naïve patients. The detection rates of CLR-resistant isolates were higher than those of CLR-sensitive isolates in both two groups, regardless of eradication treatment. CLR-resistant isolates were dominant in the corpus in eradication-failure patients, while CLR-resistant isolates were evenly distributed among the different stomach regions in treatment-naïve patients (Fig. 2b).

**Table 4:** Distribution of isolates in stomach regions and MIC values of resistant isolates from drug-heteroR/S-patients and drug-homoR-patients. Parentheses depict the number of isolates.

Drug-heteroR/S-patients			MIC^a^	Drug-homoR-patients		MIC^a^
Clinical isolates	Sensitive	Resistant		Clinical isolates	Resistant	
CLR						
Total (65)	23	42		Total (345)	345	
Antrum (30)	16	14	12	Antrum (165)	165	16
Corpus (29)	7	22	24	Corpus (168)	168	16
Tumor (6)	0	6	24	Tumor (12)	12	16
Failure (36)	12	24		Failure (280)	280	
Antrum (18)	10	8*	2.5^**^	Antrum (136)	136	24
Corpus (18)	2	16*	48	Corpus (138)	138	24
				Tumor (6)	6	28
Naïve (29)	11	18		Naïve (65)	65	
Antrum (12)	6	6	18^**^	Antrum (29)	29	12
Corpus (11)	5	6	24	Corpus (30)	30	12
Tumor (6)	0	6	24	Tumor (6)	6	10
AMX						
Total (48)	31	17		Total (12)	12	
Antrum (24)	12	12*	0.25	Antrum (6)	6	0.75*
Corpus (24)	19	5*	0.25	Corpus (6)	6	0.25*
Tumor (0)	0	0	0	Tumor (0)	0	0
Failure (48)	31	17		Failure (12)	12	
Antrum (24)	12	12*	0.25	Antrum (6)	6	0.75*
Corpus (24)	19	5*	0.25	Corpus (6)	6	0.25*
Naïve (0)	0	0		Naïve (0)	0	
MNZ						
Total (175)	120	55		Total (184)	184	
Antrum (71)	51	20	32*	Antrum (94)	94	256
Corpus (86)	61	25	48*	Corpus (90)	90	256
Tumor (18)	8	10	16	Tumor (0)	0	
Failure (54)	40	14		Failure (184)	184	
Antrum (24)	18	6	16*	Antrum (94)	94	256
Corpus (30)	22	8	160*^,^**	Corpus (90)	90	256
Naïve (121)	80	41		Naïve (0)	0	
Antrum (47)	33	14	64*^,^**			
Corpus (56)	39	17	32*^,^**			
Tumor (18)	8	10	16			
STFX						
Total (184)	61	123		Total (82)	82	
Antrum (76)	22	54	0.5	Antrum (40)	40	0.5
Corpus (78)	30	48	0.5	Corpus (42)	42	0.5
Tumor (30)	9	21	0.5	Tumor (0)	0	
Failure (90)	33	57		Failure (82)	82	
Antrum (42)	14	28	0.5	Antrum (40)	40	0.5
Corpus (42)	19	23	0.5	Corpus (42)	42	0.5
Tumor (6)	0	6	0.5	Tumor (0)	0	
Naïve (94)	28	66		Naïve (0)	0	
Antrum (34)	8	26	0.5			
Corpus (36)	11	25	0.5			
Tumor (24)	9	15	0.5			

^a^The median values (µg/mL) of resistant isolates. *CLR* clarithromycin, *AMX* amoxicillin, *MNZ* metronidazole, *STFX* sitafloxacin, *Failure* primary eradication-failure, *Naïve* treatment-naïve.

*Significant difference (*p* < 0.05), between antrum and corpus.

**Significant difference (*p* < 0.05), between primary eradication-failure and treatment-naïve.

The median MIC values of the 24 CLR-resistant isolates in eradication-failed patients were 2.5 and 48 μg/mL in the antrum and corpus, respectively. The MICs of 18 CLR-resistant isolates in treatment-naïve patients were 18, 24 and 24 μg/mL in the antrum, corpus, and tumor, respectively (Table 4). These findings indicated that CLR-resistant isolates with high MIC value emerged to become dominant in the corpus of eradication-failed patients.

We similarly analyzed 48 isolates, including 17 AMX-resistant and 31 AMX-sensitive isolates, from all 4 AMX-heteroR/S-patients that belonged to the eradication-failure group. AMX-resistant isolates dominated in the antrum. The median MIC values of these 17 AMX-resistant isolates were 0.25 and 0.25 μg/mL in the antrum and corpus, respectively. Interestingly, this finding contrasted with CLR resistance in primary eradication-failure patients.

We analyzed 175 isolates, including 55 MNZ-resistant and 120 MNZ-sensitive isolates, from all 14 MNZ-heteroR/S-patients, including 5 eradication-failed patients and 9 treatment-naïve patients. MNZ-resistant isolates were 26% and 34% of all isolates in eradication-failed patients and treatment-naïve patients, respectively. The MNZ-resistant isolates were evenly distributed across all stomach regions in the two groups. The median MICs of MNZ-resistant isolates were high for those from the corpus and antrum of eradication-failed patients and treatment-naïve patients, respectively.

We analyzed 184 isolates, including 123 STFX-resistant and 61 STFX-sensitive isolates, from all 13 STFX-heteroR/S-patients consisting of 7 eradication-failed patients and 6 treatment-naïve patients. STFX-resistant isolates were 63% and 70% of all isolates in eradication-failed patients and treatment-naïve patients, respectively. The STFX-resistant isolates were evenly distributed among the different stomach regions with similar median MIC values in the two groups.

Next, we compared the MIC values of drug-resistant isolates between drug-heteroR/S-patients and drug-homoR-patients (Table 4). CLR MIC values were highest in the isolates from the corpus in CLR-heteroR/S-patients of the eradication-failure group. AMX MIC values were highest in the isolates from the antrum of AMX-homoR-patients of the eradication-failure group. MNZ MIC values were higher in MNZ-homoR-patients than in MNZ-heteroR/S-patients for the two groups. STFX MIC values did not differ between STFX-heteroR/S-patients and STFX-homoR-patients of the two groups.

### Mutations in genes involved in CLR and AMX resistance

#### Nucleic acid analysis of *23S rRNA* with respect to CLR resistance

We randomly selected isolates to investigate mutations in *23S rRNA* related to CLR resistance. A total 116 isolates from 10 patients consisting of all 5 CLR-heteroR/S-patients, 2 CLR-homoR-patients and 3 CLR-homoS-patients were analyzed. Sixty-six CLR-resistant and 50 CLR-sensitive isolates were subjected to sequencing. CLR resistance-related point mutations in *23S rRNA*, A2142G or A2143G, were detected in 65 of 66 CLR-resistant isolates (98%). A2143G was found in 2 of 50 CLR-sensitive isolates. A2142G and A2143G were detected in 5% (3/66) and 94% (62/66) of CLR-resistant isolates, respectively. Next, A1738G mutation was found in 44% (29/66) of CLR-resistant isolates, but only one of 50 CLR-sensitive isolates. Furthermore, A1738G was found in 47% of 62 CLR-resistant isolates harboring A2143G. There was no CLR-resistant isolate harboring single A1738G mutation, leading to the relationship between A1738G and A2143G. We therefore compared the MIC values between 33 isolates with A2143G and 29 isolates with A1738G and A2143G. The median MICs were 32 μg/mL for A2143G and 16 μg/mL for A1738G and A2143G, indicating reduced MICs for isolates with the two mutations.

#### Amino acid analysis of PBP1A with respect to AMX resistance

**Figure 3:**
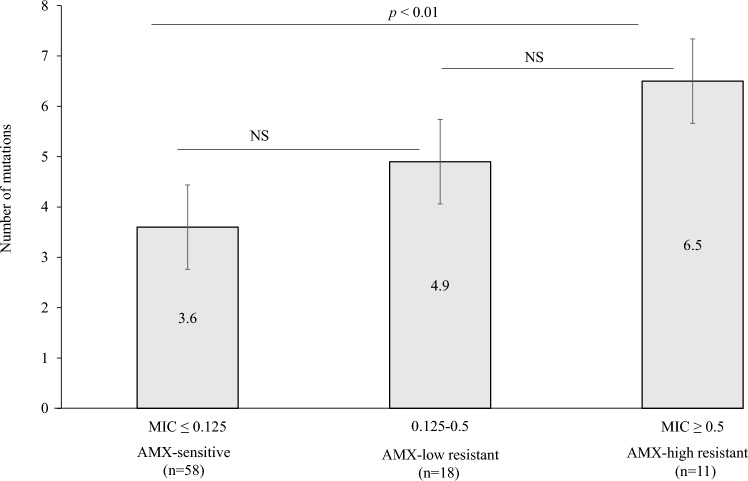
Correlation between the MIC of AMX (μg/mL) and the number of mutations in penicillin-binding protein 1A. *NS* not significant, *AMX* amoxicillin.

A total of 87 isolates from 8 patients consisting of all 4 AMX-heteroR/S-patients, 1 AMX-homoR-patient, and 3 AMX-homoS-patients were subjected to sequencing. Among the 87 isolates, 29 were AMX-resistant and 58 were AMX-sensitive. Seventeen mutations resulting in amino acid changes were detected in the 87 isolates and were categorized into three MIC levels: AMX-sensitive (≤ 0.125), AMX-low resistance (AMX^LR^) (0.125–0.5) and AMX-high resistance (AMX^HR^) (≥ 0.5) (Table 5). Among the 17 mutations, two, A480V and G591K, were observed in AMX^HR^ only. We therefore evaluated MIC values in recombinant strains that possess these two mutations. In addition, the number of mutations detected was evaluated for association with the three levels of AMX resistance. A statistically significant difference (*p* < 0.01) was observed between AMX-sensitive and AMX^HR^ isolates (Fig. 3), indicating that the increased number of mutations contributes to the increased MIC value of AMX.

**Table 5:** Amino acid substitutions in PBP1A of 87 isolates from 8 patients.

Amino acid position	Change of amino acid	AMX^S^	AMX^LR^	AMX^HR^
		(n = 58)	(n = 18)	(n = 11)
374	V374L	11	1	0
414	S414R	3	5	4
469	V469M	11	0	0
480^a^	A480V	0	0	4
481	A481V	10	2	0
504	N504D	58	18	11
515	I515M	10	7	7
556	T556S	13	7	4
562	N562Y	0	5	7
589	S589G	58	18	11
591^a^	G591K	0	0	6
593	T593G	0	5	7
	T593S	7	5	0
	T593A	11	1	0
595	G595S	0	6	7
595_596	595_596 Insertion G	10	9	4
599	A599T	8	0	0

AMX^S^, Amoxicillin-sensitive (MIC ≤ 0.125 µg/mL).

AMX^LR^, Amoxicillin-low resistant (MIC 0.125–0.5 µg/mL).

AMX^HR^, Amoxicillin-high resistant (MIC ≥ 0.5 µg/mL).

^a^Mutations found are unique to only AMX^HR^ isolates.

#### Evaluation of AMX and CLR MIC values using *H. pylori* recombinant strains

Six recombinants based on three AMX-sensitive *H. pylori* strains were produced by homologous recombination using two plasmids harboring either PBP1A G591K or A480V mutations. E7.J99 and E50.J99 recombinants strains were produced from strain J99 and possessed either G591K or A480V mutations, respectively. We performed drug-susceptibility tests with clones from all six recombinants and showed that the MIC values of all six recombinants were obviously increased (Table 2). We also showed that the MIC value of recombinants with G591K was higher than that for recombinants with A480V. The G591K mutation more effectively converted the AMX-sensitive strain to the AMX-resistant strain compared with the A480V mutation. We similarly prepared six more recombinants possessing the same amino acid sequences but without G591K and A480V. Drug-susceptibility tests demonstrated the MIC values of all six recombinants to be slightly increased but not up to the level of AMX-resistance. Taken together, these findings confirmed the G591K and A480V mutations to be associated with AMX resistance. We also assessed whether the A1738G mutation contributes to CLR resistance using recombinant strains derived from CLR-sensitive strain J99 lacking the A1738G mutation. The recombinants in which A1738G was replaced were confirmed by PCR and sequencing. Drug-susceptibility tests with these recombinants revealed their MICs to be unchanged compared with the strain J99 lacking the A1738G mutation.

## Discussion

The failure to eradicate *H. pylori* infection with common antibiotics is increasing because of drug-resistant isolates. *H. pylori* has high genetic diversity and produces a community containing many mutants, which adapt to the microenvironments of an individual’s stomach to achieve persistent infection. However, single isolate is usually used in studies concerned with *H. pylori* infection and for clinical assessment. Prevalence of heteroresistance (drug-heteroR/S) to antibiotics is a multifaced issue for clinical practices and research. In a limited number of literatures, the heteroresistance rates between studies are difference and still controversial. The different rates across diverse populations and geographical regions emphasize the need of methodology to understand the exact situation of *H. pylori* in the stomach^37,41^. Furthermore, patients’ information such as eradication therapy and manifestation is unclear in previous reports. In this study, we used multiple isolates from individual patients to determine the community heterogeneity in individual stomachs. We showed that the rate of heteroresistance was 12%, 10%, 33% and 31% for CLR, AMX, MNZ, and STFX, respectively. The heteroresistance rate is higher than those of previous reports. In addition, the rates differed between primary eradication-failure and treatment-naïve, and within gastric regions of the stomach. These provided important insight into the characteristics of *H. pylori* infection for clinical practices.

Among the 42 patients examined, 64% were drug-heteroR/S for at least one of the four drugs tested, and these rates were 54% and 86% in the 28 primary eradication-failed patients and 14 treatment-naïve patients, respectively. This indicates that *H. pylori* community heterogeneity is more common than expected. Among the 28 patients in whom primary eradication-failed using CLR and AMX, there were 11%, 14%, 18% and 25% drug-heteroR/S-patients for CLR, AMX, MNZ and STFX, respectively. The prevalence of drug-heteroR/S-patients was lower for CLR and AMX compared with MNZ and STFX. Among the 14 treatment-naïve patients, there were 14%, 0%, 64% and 43% drug-heteroR/S-patients for CLR, AMX, MNZ and STFX, respectively. The prevalence of drug-heteroR/S-patients was higher among treatment-naïve patients for CLR, whereas for AMX, it was higher among primary eradication-failed patients. However, interestingly, CLR-homoR-patients and AMX-homoS-patients predominated among the primary eradication-failed patients. In contrast, MNZ-heteroR/S-patients were the most common among the treatment-naïve patients. In this study, most treatment-naïve patients had malignancies, such as gastric cancer and mucosa-associated lymphoid tissue lymphoma, indicating an effect of the cancerous stomach microenvironment on MNZ resistance. The *rdxA* and *frxA* genes are responsible for the activity and function of MNZ within bacteria^42^. In stomachs with a cancerous lesion, stresses in microenvironments, such as oxidation, inflammation, and methylation, affect bacterial ecology^43,44^. Epigenetic analysis showed considerably more methylation of the *rdxA* gene in cancer than in non-cancer tissue^45^. Investigation of a greater number of cancer patients with and without primary eradication therapy is needed.

We analyzed the characteristics of drug-resistant isolates in drug-heteroR/S-patients. In the 28 primary eradication-failed patients, the proportion of CLR-resistant isolates and their MIC values were higher in the corpus than in the antrum. Conversely, AMX-resistant isolates were dominant in the antrum. However, no differential distribution between the antrum and corpus was found in MNZ-heteroR/S-patients and STFX-heteroR/S-patients. These findings indicated that CLR-resistant and AMX-resistant isolates were preferentially distributed in different stomach regions and may emerge after primary eradication failure. Communities of *H. pylori* strains with various characteristics can eventually establish persistent infection in different gastric microenvironments depending on within-host evolution^46^. Examining two isolates from one patient enabled the different characteristics of isolates colonizing the antrum and corpus to be observed^47^. However, the study of two isolates cannot accurately assess drug-heteroR/S-patients with coexisting resistant and sensitive isolates. This study of multiple isolates provides insights for understanding the relationship between gastric regions and drug-resistant isolates in primary eradication-failed patients in which resistant and sensitive isolates coexist with niche-specific adaptation. A precise understanding of the drug-susceptibility status of colonized *H. pylori* in patients who have failed primary eradication will promote effective eradication strategies for next-generation therapeutics. This should be considered in research and clinical assessments of *H. pylori* infection.

The prevalence of CLR-resistant strains remains a concerning issue worldwide. Various regions have reported primary resistance rates of 15–40% or higher. In Japan, the primary CLR resistance rate has gradually increased in recent decades, rising from 7 to 48%^28,48^. These data are from the examination of one or two isolates from treatment-naïve patients. In our study with multiple isolates from 14 treatment-naïve patients, the proportion of patients infected with CLR-resistant strains (CLR-homoR-patients and CLR-heteroR/S-patients) was 50%, consistent with the primary resistance rate above. However, of the 28 patients who failed primary eradication, 97% were infected with CLR-resistant strains, exceeding the highest global average (67%) reported using two or three isolates^49^. Our study with multiple isolates indicates that CLR-resistant strains emerged in most patients in whom primary eradication had failed, resulting in the predominance of CLR-homoR-patients rather than CLR-heteroR/S-patients. In contrast, AMX-heteroR/S-patients predominated over AMX-homoR-patients. This indicated that *H. pylori* may not convert to AMX resistance, even if primary eradication fails.

Mutations in the *H. pylori 23S rRNA* gene reduce affinity to CLR and significantly increase MICs^50^. Two mutations in *23S rRNA*, A2142G and A2143G, are related to CLR resistance^51^. We also showed that A2143G is associated with CLR resistance. We found the A1738G mutation to be in 44% of CLR-resistant isolates (29/66) and in 2% of CLR-sensitive isolates (1/50). The CLR resistance mutation, A1738G, was analyzed using recombinant strains prepared using CLR-sensitive strain J99 lacking the A1738G mutation. The MICs of the recombinants possessing A1738G were unchanged, indicating that the A1738G mutation has little effect on CLR resistance. We then found a relationship between A1738G and A2143G mutations in CLR-resistant isolates. The mean MIC for A2143G was 54 μg/mL and for A1738G plus A2143G was 20 μg/mL. The CLR-resistant isolates harboring the two mutations of A1738G and A2143G had a reduced MIC, compared to the isolates harboring A2143G. These indicated that adding A1738G to the CLR-resistant isolates harboring A2143G may lead to lower a MIC value^52^. This study also found A2143G in two CLR-sensitive isolates with MICs of 0.75 and 0.5 µg/mL, close to ≥ 1 μg/mL. We reexamined MICs of the two strains after they had been frozen for 1 year and demonstrated CLR resistance. Therefore, *H. pylori* with A2143G and close to the MIC (≥ 1 μg/mL) should be treated as a resistant strain in clinical practice. A newly developed detection kit is expected to be useful in clinical practice for rapid and accurate identification of 2142/2143 mutations.

AMX resistance in *H. pylori* is caused by modifications to penicillin-binding proteins, particularly PBP1A, which prevent the binding of AMX to the cell wall. The primary AMX resistance rates range from approximately 3.48–9.5% and are increasing globally^53–55^. We demonstrated the AMX resistance rate in 28 primary eradication-failed patients to be 18%, higher than the global average reported in primary eradication-failed patients^49,56^. Of the 28 primary eradication-failed patients, only 4% were AMX-homoR-patients, while 14% were AMX-heteroR/S-patients. Therefore, this study with multiple isolates indicates that drug-susceptibility testing using multiple isolates is more reflective of actual infection.

Seventeen amino acid mutations were detected in 87 isolates, including in all 29 AMX-resistant and 58 AMX-sensitive isolates. Various amino acid substitutions in PBP1A that affect AMX resistance have been previously reported, such as S414R, N562Y, T593A, G595S, and A599T^26,28,57^. N562Y and G595S were found in an AMX-resistant isolate, and S414R and T593A were found in both AMX-resistant and AMX-sensitive isolates. A599T was not detected in any of the 29 AMX-resistant isolates, consistent with a previous report from Japan^28^. Interestingly, G591K was detected in 55% (6/11) of AMX^HR^ isolates with MICs ≥ 0.5 μg/mL, but in 0% of AMX-sensitive and AMX^LR^ isolates. A480V was also detected in 36% (4/11) of AMX^HR^ isolates, but not in AMX-sensitive or AMX^LR^ isolates. Therefore, these mutations are considered to be associated with AMX resistance and high MIC values. Kageyama et al*.* revealed the presence of A480V in 1 of 49 AMX-sensitive and 2 of 8 AMX-resistant isolates with unknown MICs^28^. Kuo et al. showed G591R to only be present in AMX-resistant isolates^58^. We cannot exclude the possibility that these mutations are strain-specific polymorphisms. Therefore, we prepared recombinants based on three AMX-sensitive strains harboring G591K or A480V and subjected them to AMX-susceptibility testing. All six recombinants had increased MICs and reached AMX resistance levels; only E50.HPK5 carrying A480V failed to reach the resistance level. The recombinants harboring G591K acquired higher MICs compared with those harboring A480V, indicating that G591K affects the induction of AMX-resistance in *H. pylori*. Additionally, to confirm the contribution of the region surrounding G591K or A480V to the MIC values, we prepared six additional recombinants without G591K or A480V. The additional recombinants included the recombinants harboring N562Y or S414R. The MIC values of all six recombinants were unchanged, indicating that G591K and A480V are essential for the increased MICs of AMX. N562Y, T593G, and G595S in PBP1A are located near the penicillin-binding pocket^59^; therefore, G591K may interfere with the affinity of AMX for the PBP1A. This study of recombinants directly demonstrated that at least two mutations in PBP1A, G591K and A480V, are associated with AMX resistance. However, A480V is present in 1 of 49 AMX-sensitive isolates^28^. The other amino acid sequences may mitigate the effect of A480V on AMX susceptibility, resulting in lower MICs^52^. The complex relationship between genetic mutations and therapeutic efficacy indicates the continued importance of monitoring and further research to understand the underlying mechanisms driving resistance development.

This study showed the high prevalence of heteroresistance in stomach with multiple isolates. We propose that the mix sample consisting of several isolates obtained during the primary selection step could be useful for cost-effective and labor-friendly to susceptibility testing in the clinical laboratory. Recently, it has become possible to detect the CLR-resistant 2142/2143 mutation using bulk DNA extracted from gastric juice by Japanese health insurance. In the future, hot-spot nucleotide positions associated with each antibiotic resistance will be identified, leading to the development of such detection methods can be used in clinical laboratory.

We performed drug-susceptibility testing of multiple isolates to clarify the heterogeneity of the *H. pylori* community in the stomach. Testing with multiple isolates helps to reveal the precise drug-susceptibility status of *H. pylori* colonizing different gastric regions in individual patients, especially in primary eradication-failure patients. Such testing reflects the current state of *H. pylori* infection and can inform improved eradication strategies.

This study has the limitation as below. Of the forty-two patients participated in this study, fourteen patients were treatment-naïve. Of those, eight patients were suffered from malignancies, and such situations could affect the MIC values. Therefore, the number of treatment-naïve patients with and without malignancy will need to be increased to clarify such effects. In addition, the susceptibility test for only STFX was performed by agar dilution method due to no STFX strip. Although we are confident in the susceptibility results, detailed MIC values might to be difficult to combine for all MIC values for four antibiotics. We carefully obtained multiple isolates in the primary selection step, however, the possibility that several isolates originated from a single colony was not completely excluded. Finally, we validated the mutations using recombinants prepared, however, it could not exclude that other mutations affect the MIC value more or less.

## Conclusions

This study of multiple isolates from individual patients demonstrates the coexistence of drug-resistant and drug-sensitive *H. pylori* isolates in the stomach and their drug susceptibility status. Drug-resistant isolates with different MIC values preferentially colonized different stomach regions of patients for whom primary eradication failed. Preparation and analysis of recombinants, enabled us to show that G591K and A480V in PBP1A are essential mutations associated with AMX resistance.

### Supplementary Information

Supplementary Information.

## Data Availability

The datasets used and analyzed during the current study are available from the corresponding author on reasonable request.
