# Supplementary material for: Evidence of Helicobacter pylori heterogeneity in human stomachs by susceptibility testing and characterization of mutations in drug-resistant isolates
Source: Sci Rep. 2024 May 27;14:12066. doi: 10.1038/s41598-024-62200-1 (PMC11130178; doi:10.1038/s41598-024-62200-1)
Supplement: Supplementary file 1 — Supplementary Information. [file 41598_2024_62200_MOESM1_ESM.pdf]

Evidence of *Helicobacter pylori* heterogeneity in human stomachs by susceptibility testing and characterization of mutations in drug-resistant isolates

Jahirul Md Islam[1], Yukari Yano[2], Aoi Okamoto[1], Reimi Matsuda[1], Masaya Shiraishi[1], Yusuke Hashimoto[1], Nanaka Morita[1], Hironobu Takeuchi[3], Narufumi Suganuma[4], Hiroaki Takeuchi[1]*

*, corresponding author

Email: htake@iuhw.ac.jp

Target gene	Primer name	Primer sequence (5´- 3´)	PCR condition	Cycle	Amplicon size (bp)	Source
16S rRNA	JW22	CGTTAGCTGCATTACTGGAGA	2min at 96˚C; 30sec at 96˚C,	35	295	38
	JW23	GAGCGCGTAGGCGGGATAGTC	30sec at 60˚C, 30sec at 72˚C			
23S rRNA	CAM 23S-HP1	TTGGAGGGAAGGCAAATCCAC	2min at 96˚C; 30sec at 96˚C,	40	1093	39
	CAM 23S-HP2	ACGTTCTGAACCCAGCTCGC	30sec at 56˚C, 50sec at 72˚C			
23S rRNA	CAM23SHP-2F	GAGGTTGGCTTAGAAGCAGC	2min at 96˚C; 30sec at 96˚C,	40	916	This study
	CAM23SHP-2R	GACAGCTCCCATCTCGTTAC	30sec at 56˚C, 50sec at 72˚C			
A1738G in	CAM23SHP-2F	GAGGTTGGCTTAGAAGCAGC	2min at 96˚C; 30sec at 96˚C,	35	595	This study
23S rRNA	CAM 23SHP-3Rm	GCGCCTTAGAATACTCATCC	30sec at 67.5˚C, 50sec at 72˚C			
A1738A in	CAM23SHP-2F	GAGGTTGGCTTAGAAGCAGC	2min at 96˚C; 30sec at 96˚C,	35	595	This study
23S rRNA	CAM 23SHP-3Rn	GCGCCTTAGAATACTCATCT	30sec at 67˚C, 50sec at 72˚C			
pbp1A	PBP1(Hp)-F	GCATGATCGTTACAGACACG	2min at 94˚C; 30sec at 94˚C,	35	905	28
	PBP1(Hp)-R	ATCCACGATTTCTTTACGC	30sec at 60˚C, 1min at 72˚C			
pbp1A	PBP1-2F	ATATCGCTCCCTATGTCGTG	2min at 94˚C; 30sec at 94˚C,	35	936	This study
	PBP1-2R	AAGCCAATGAACCAAGCGTC	30sec at 60˚C, 1min at 72˚C			
pCR™2.1-TOPO®	M13-F	GTAAAACGACGGCCAG	2min at 94˚C; 30sec at 94˚C,	35	1108 including 905	This study
	M13-R	CAGGAAACAGCTATGAC	30sec at 52˚C, 1min at 72˚C		fragment	

Supplementary Table S1. Primers and PCR conditions used in this study.
